# The Effect of Width-Mismatch of Modulated Nanowaveguides on the Thermoelectric Efficiency

**DOI:** 10.3390/mi14101912

**Published:** 2023-10-07

**Authors:** Antonios-Dimitrios Stefanou, Xanthippi Zianni

**Affiliations:** Department of Aerospace Science and Technology, School of Science, National and Kapodistrian University of Athens, 34 400 Psachna, Greece; antstefanou@aerospace.uoa.gr

**Keywords:** nanowaveguides, width-modulated nanowires, quantum dot, phonon conduction, electron conduction, thermoelectric efficiency

## Abstract

Width-modulated nanowaveguides are promising for thermoelectric efficiency enhancement because electron and phonon transport properties can be geometrically tuned for improved performance. The shape of the modulation profile drastically affects the transport properties. Optimization of the width modulation for simultaneous maximum thermoelectric transport and minimum thermal transport is challenging because of the interconnected electron and phonon transport properties. We addressed this problem by analysing the effect of each characteristic dimension of a single rectangular modulation unit on electron and phonon transport. We identified distinct behaviours for electrons and phonons. We reveal that whereas phonon thermal conductance decreases with increasing width-mismatch, the electron thermoelectric power factor shows a non-monotonic dependence. It is pointed out that optimal width-mismatch that maximizes thermoelectric efficiency is mainly determined by electron transport and should be identified by maximizing the thermoelectric power. Our work points to a new strategy of optimizing geometry-modulated metamaterials for maximum thermoelectric efficiency.

## 1. Introduction

Thermoelectric (TE) energy conversion aims to address the current need of our society for low-cost green energy production and waste heat recycling. Low-efficiency traditional thermoelectric materials prohibited widespread thermoelectric applications for long time. The efforts of the thermoelectric community have concentrated on alternative approaches, such as using low-dimensional and nanostructured materials, to enhance the TE efficiency. Much progress has been achieved and it remains an active research area. Additional interest in thermoelectrics emerged from the potential of TE materials to power the Internet of Things. Traditional TE materials are not suitable for integration into microelectronics industrial processes. Novel strategies to enhance the TE efficiency of technologically important materials are of predominant importance and interest. Thermoelectric metamaterials such as width-modulated nanowaveguides with enhanced TE properties can contribute to the present tasks of the research on thermoelectric energy conversion [1]. Their operation is based on the same principles as the low-dimensional and nanostructured materials that revolutionized science and technology, providing the ability to engineer the energy band structure and properties of matter with superlattices of different materials [2,3,4]. In both classes of metamaterials, the good TE properties originate from (i) quantum confinement and specifically the sharp propagation thresholds of the low-dimensional density of states (DOS) and (ii) enhanced phonon scattering at geometrical and/or compositional discontinuities [5,6,7,8,9,10,11].

The measure of TE efficiency is the dimensionless figure of merit ZT defined as ZT = σS^2^T/(k_ph_ + k_e_), where σ is the electrical conductivity, S is the Seebeck coefficient, and k_ph_ and k_e_ are the phonon and electron thermal conductivities, respectively. A good TE material should be a bad heat conductor (with low k_ph_ and k_e_) to minimize thermal leakage from the hot to the cold contact. Moreover, it should have good electrical conductivity and a high Seebeck coefficient to provide high TE power (high σS^2^). Typically, electron and phonon transport properties are interconnected and mechanisms that decrease phonon thermal conduction also decrease electron conduction and deteriorate TE power. This problem has been encountered in low-dimensional material structures and is also present in geometry-modulated NW [1]. To deal with this, optimizing disorder in the modulation profile was proposed [12]. It was shown that the modulation shape drastically affects electron and phonon transport [10,11]. Disorder-induced enhancement of the thermoelectric efficiency of width-modulated nanowires was theoretically demonstrated [12]. It was shown that an aperiodic modulation profile decreases phonon conduction below the periodic modulation value and that optimal aperiodic modulation could preserve high TE power and low thermal conduction [12]. It has become evident that electron and phonon transport properties can be optimized by designing aperiodic geometry modulation.

Geometry modulation can be realized in multiple ways and with variable degrees of complexity. Designing geometry modulation for optimal thermal and electric transport is challenging. Physics can provide valuable guidance to this task. In the case of phonon transport, a physics rule was revealed: phonon conduction decreases monotonically with increasing disorder in the width-modulation profile of aperiodic nanowaveguides [11,12,13]. Optimal width modulation for minimum disorder was found for maximum disorder in the modulation profile, i.e., maximum number of non-identical modulation units (quantum dots). Identifying a physics rule for optimizing width modulation for maximum TE efficiency remains an open question. It requires further understanding of the effect of width-mismatch on phonon and electron transport. Here, we address this problem and find interesting new evidence. We analysed the effect of each characteristic dimension of a single modulation unit on electron and phonon transport. We report on distinct behaviours for electrons and phonons. We show that whereas phonon thermal conductance decreases with increasing width-mismatch, the electron thermoelectric power factor shows a non-monotonic dependence. Our calculations support that the optimal width-mismatch that maximizes TE efficiency is mainly determined by electron transport and should be identified by maximizing the TE power. The structures of interest and the theoretical model are described in Section 2. Our results are presented and discussed in Section 3. Our main conclusion is drawn in Section 4.

## 2. System and Theoretical Model

**Figure 1:**
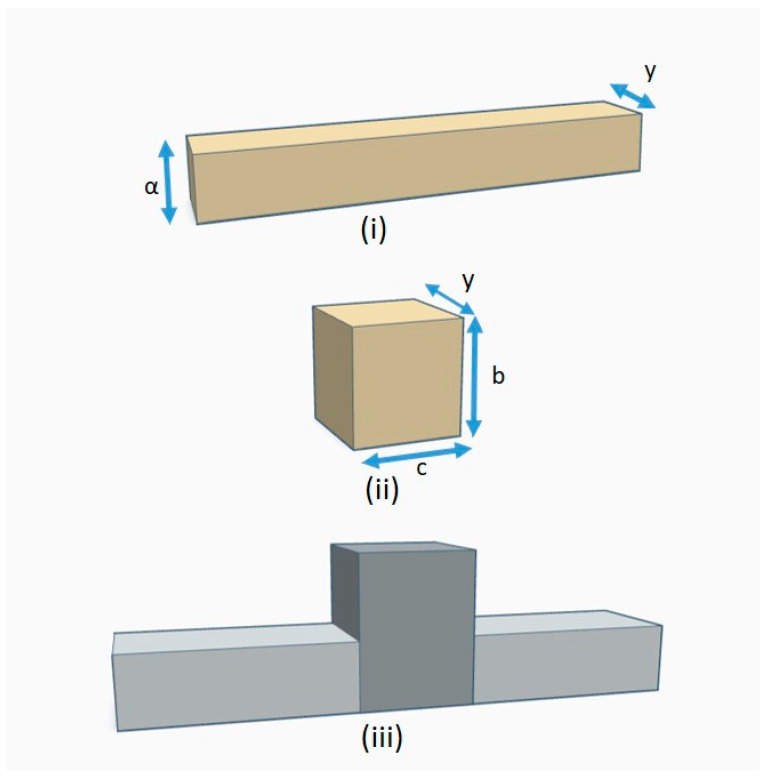
Schematic representation of the investigated ΜΝW: (**i**) uniform NW of width α and depth y, (**ii**) modulation unit (QD) with width b, length c, and depth y. (**iii**) The MNW emerges from the coupling of the NW and the QD.

We consider a width-modulated nanowaveguide (MNW) consisting of an infinite nanowire (NW) modulated by a single modulation unit referred to as a quantum dot (QD). A schematic diagram of the structure is shown in Figure 1iii as emerging from the coupling between the NW and the QD. The actual form has been chosen because it is the simplest representative geometry that captures physics [1]. We present representative results for GaAs NWs of width a = 10 nm and depth y = 12 nm modulated by a QD with variable width b and length c.

We work in the ballistic transport regime, which is suitable for studying the effects of geometry modulation on electron and phonon states and transport unscreened by effects due to additional scattering mechanisms [14,15]. We use Landauer formalism for the transport of electrons and phonons in terms of the transmission coefficient [10,12,16,17,18,19], which is calculated using scattering matrix theory [20]. We keep our study at low temperatures to avoid thermal broadening of quantum confinement effects on transport [21,22].

The electron conductance G, the Seebeck coefficient S, and the thermal coefficient K are, respectively, given by the following expressions [14,23]:

(1) $$G=-\frac{2e^{2}}{h}\int dET_{e}(E)\frac{\partial f}{\partial E}$$

(2) $$S=-\frac{1}{eT}\frac{\int dE(E-E_{F})T_{e}(E)\frac{\partial f}{\partial E}}{\int dET_{e}(E)\frac{\partial f}{\partial E}}$$

(3) $$K=\frac{2e^{2}}{h}\frac{1}{e^{2}T}\int dE{\left(E-E_{F}\right)}^{2}Te(E)\frac{\partial f}{\partial E}$$

where T_e_(E) stands for the electron transmission coefficient and E_F_ for the electron Fermi energy.

The electron thermal conductance k_e_ is calculated in terms of the above coefficients using the expression

(4) $$\kappa_{e}=-K-S^{2}GT$$

In plots, the conductance is expressed in the unit of the conductance quantum 2 e^2^/h and the thermal conductance in the unit of the thermal conductance quantum κ_0_ = π^2^k_b_^2^/3h.

The phonon thermal conductance k_ph_ is given in terms of the phonon transmission coefficient with the following expression [24,25,26]:

(5) $$\kappa_{ph}=\frac{h^{2}}{k_{B}T}\sum_{m}\frac{1}{2\pi }\int_{\omega_{m}}^{\omega }{Τ}_{m}(\omega )\frac{\omega^{2}e^{hbar\omega /k_{Β}Τ}}{(e^{\frac{hbar\omega }{k_{Β}Τ}}-1{)}^{2}}d\omega$$

where ω_m_ is the cut-off frequency of the m mode and T_m_(ω) is the transmission coefficient for phonon mode m and phonon frequency ω [27,28,29,30]. Every propagation mode contributes with each frequency to the integration and the total transmission coefficient of the phonons can be calculated from the sum over all phonon modes m:

(6) $$T_{ph}\left(\omega \right)=\sum_{m}T_{m}(\omega )$$

In the plots, the phonon frequency ω is expressed in units of Δ, where Δ = ω_m+1_ − ω_m_ = πu/d is the splitting of the cut-off frequency between the m + 1 and the m mode, d is the width of the ΝW, and u is sound velocity. Τhe phonon thermal conductance is expressed in the unit of the quantum thermal conductance κ_0_ = π^2^k_b_^2^/3 h.

The thermoelectric efficiency of the nanostructures is estimated using the dimensionless figure of merit ZT that is defined as

(7) $$ZT=\frac{S^{2}GT}{\kappa }$$

where the numerator GS^2^T is the thermoelectric power factor, TPF, and the denominator κ is the total thermal conductance that consists of two contributions, the electron and phonon thermal conductivities:

(8) $$\kappa =\kappa_{e}+\kappa_{ph}$$

An optimal figure of merit ZT_0_ can be defined by neglecting the phonon thermal conductance. Then, the figure of merit ZT can be expressed as [10]

(9) $$ZT=ɑZT_{0}$$

(10) $$ɑ=(1+\frac{\kappa_{ph}}{\kappa_{e}}{)}^{-1}$$

Parameter ɑ approaches 1 for negligible phonon thermal conductance.

## 3. Results and Discussion

Optimization of width-modulated nanowaveguides for maximum thermoelectric efficiency would require better understanding and optimization of the effect of width-mismatch on electron and phonon transport. To address this open problem, we investigated the effect of varying the two characteristic widths of the modulation unit, namely the QD width b and the QD length c (Figure 1).

In the quantum confinement regime, electron and phonon transport takes place within low-dimensional energy sub-bands [14]. This is directly reflected in the energy dependence of the transmission coefficient. In NWs, the transmission coefficient is a monotonic function of carrier energy and has a step-like form [23]. A step of height 1 corresponds to a propagation through a sub-band. Additional steps appear in the transmission coefficient when propagation through an additional sub-band becomes possible with increasing carrier energy. In width-modulated nanowaveguides, this structure is distorted due to wave interference at width discontinuities [10,11]. Depending on the degree of width-mismatch and the dimensions of the modulation unit, destructive interference takes place to variable extents and decreases the transmission probability below the value 1 of the perfect NW. The energy structure of the transmission coefficient becomes a non-monotonic function with characteristic fluctuations that deviate more from the step-like form as width-mismatch increases. This behaviour is generic for both type of carriers, electrons and phonons, although the geometrical length scale may have different quantitative effects on each of them. As a general trend, we can notice that conduction decreases with increasing width-mismatch due to more extended destructive interference at width discontinuities. This evolves with steeper peaks and dips and more pronounced energy fluctuations in the transmission coefficient. The Seebeck coefficient and the TPF are thereby enhanced. Interestingly, the interplay of decreasing conduction and increasing TPF shows in different ways when changing the QD width b or the QD length c. In what follows, we present and discuss our results in each case.

### 3.1. The Effect of the QD width b

**Figure 2:**
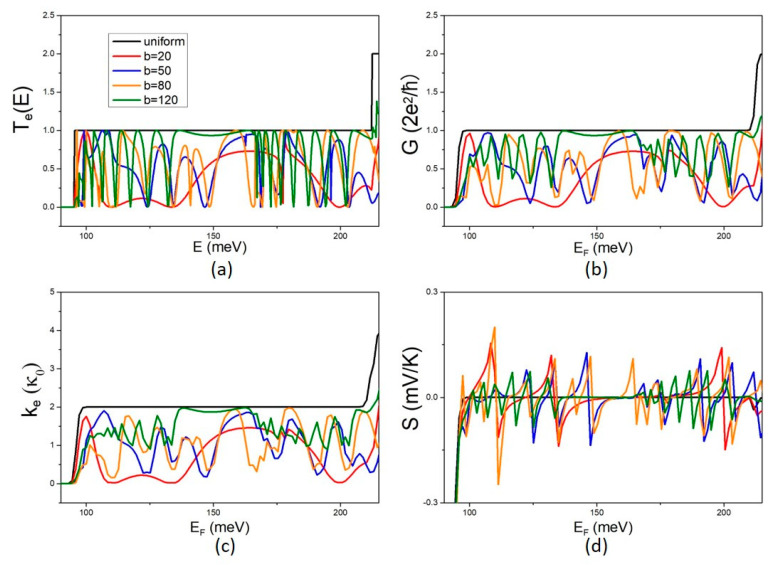
The effect of the QD width b on electron transport: (**a**) The energy dependence of the transmission coefficient T_e_(E) for the uniform NW and the MNW for different values of the QD width b. The corresponding electron conductance G, electron thermal conductance k_e_, and the Seebeck coefficient S are shown in (**b**,**c**,**d**), respectively, at T = 5 K.

The effects of the QD width b on the electron transmission coefficient T_e_(E) and the electron transport coefficients, G, k_e_, and S, are shown in Figure 2 for variations in b in the range 10 nm–120 nm and constant QD length c = 20 nm. Note that the uniform NW is for b = 10 nm irrespective of the value of parameter c.

The electron transmission coefficient T_e_(E) is zero up to a certain energy threshold and becomes finite above it. Above the threshold, kinetic energy becomes non-zero and electron transmission is possible. The threshold is determined by the NW width α and the effective mass of the constituent material (GaAs in the present case) [14]. As expected, T_e_(E) shows a step-like structure for the uniform NW and non-monotonic energy fluctuations with peaks and dips for the MNW. The peaks and dips of the transmission coefficient become denser with increasing b (Figure 2a), implying that destructive interference increases with increasing QD width b. This dependence is attributed to the increasing density of QD states with increasing b. The higher density of states of the modulating QD results in a higher number of interferences and thus more destructive interference dips. The electron conductance G and the thermal conductance k_e_ follow the energy dependence of the transmission coefficient (Figure 2b,c, respectively). The Seebeck coefficient, S, follows the energy derivative of the transmission coefficient and shows a peaked structure with higher peaks at sharper conduction thresholds [1]. Interestingly, we found that the peaks of S become higher with increasing b up to b = 80 nm and then, at b = 120 nm, they increase in density but decrease in height. This behaviour is interpreted as an effect of quantum confinement. The size of the QD increases with increasing b, and thus the density of the QD discrete energy states also increases, resulting in an increasing density of peaks in the transmission coefficient and the transport properties. This explains the higher density of S peaks with increasing b. The S peaks decrease in height because the derivative of G is smaller as the transmission coefficient thresholds are smoother when the energy spectrum is denser and coupling between states is stronger.

**Figure 3:**
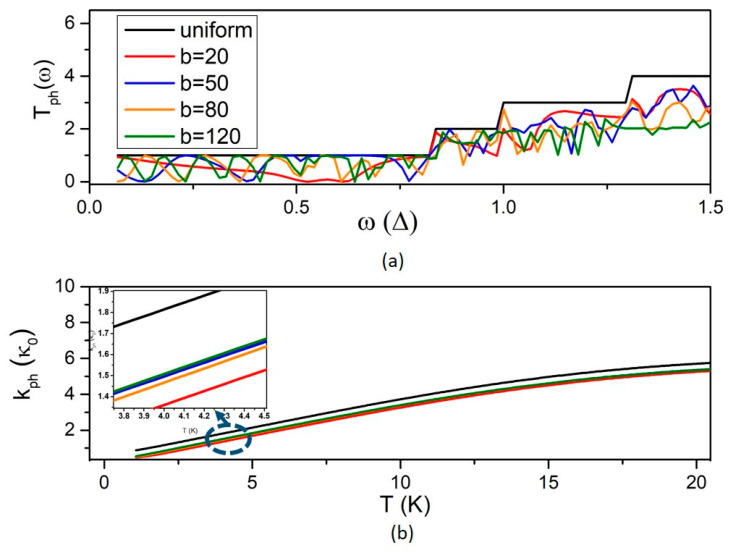
The effect of the QD width b on phonon transport. (**a**) The phonon transmission coefficient, and (**b**) the phonon thermal conductivity versus temperature T for different values of the QD width b as denoted in the legend. The inset zooms in curves for different values of b.

The phonon transmission coefficient T_ph_ is shown in Figure 3a. Similarly, as for electron waves, destructive interference of phonon waves at width discontinuities results in a decrease in the transmission coefficient below the value 1 of the uniform NW. This explains why the phonon thermal conductance of the MNW is smaller than that of the uniform NW (Figure 3b). Notably, the decrease in the phonon thermal conductance depends weakly on b and seems to saturate above b ~50 nm (see inset of Figure 3b). This is because the fluctuations in the phonon transmission coefficient become denser with increasing b up to ~50 nm but change weakly for b increasing further above this value (Figure 3a).

**Figure 4:**
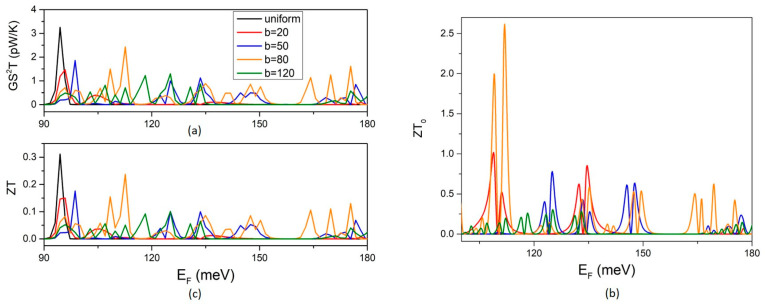
The effect of the QD width b on the thermoelectric efficiency: (**a**) the thermoelectric power factor TPF, (**b**) the optimal figure of merit ZT_0_, and (**c**) the figure of merit ZT at T = 5 K.

The TPF is determined solely by the electron transport properties. It shows pronounced peaks and dips due to the fluctuations in the electron conductance and its derivative (Figure 4a). The highest peak is shown for the uniform NW at the propagation threshold. The TPF of the MNW shows multiple peaks with lower heights than the peak of the uniform NW. The density of the TPF peaks increases with increasing width b. However, the heights of the peaks vary non-monotonically with increasing b. They increase up to b = 80 nm and then decrease at b = 120 nm. The same non-monotonic dependence is found for the optimal figure of merit ZT_o_. The decrease in the optimal TE efficiency above a certain value of b is shown even more clearly in the fluctuations in ZT_o_. The peaks are denser but clearly lower for b = 120 nm than for b = 80 nm. The high values of ZT_o_ are due to the small electron thermal conductance at the transmission coefficient dips (Figure 2c). The TE figure of merit obtains significantly lower values when phonon thermal conductance is also considered. This is shown by the lower values of ZT compared to ZT_o_ (Figure 4c). The ZT shows non-monotonic dependence on the width b similarly to the TPF. It increases with increasing b up to ~80 nm and then decreases at b = 120 nm. This is because phonon thermal conductance depends weakly on the width variation b, and thus the dependence of ZT on width b is determined by the TPF. It is concluded that the variation in ZT with b is mainly determined by electron transport. ZT shows an optimum for b in the range 80–120 nm. However, it should not be overlooked that the magnitude of the ZT itself does depend on the actual value of the phonon thermal conductance and the shape of the modulation profile [12].

### 3.2. The Effect of the QD Length c

**Figure 5:**
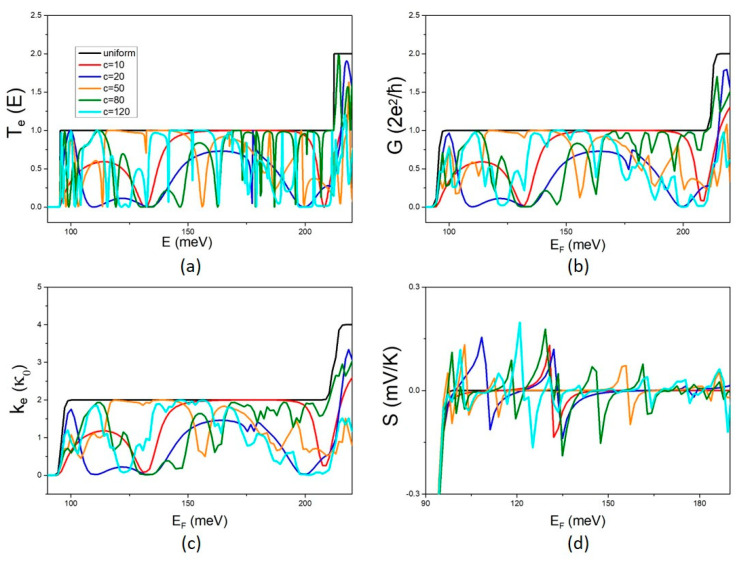
The effect of the QD length c on electron transport: (**a**) The energy dependence of the transmission coefficient T_e_(E) for the uniform NW and the MNW for different values of the QD length c. The corresponding conductance G, electron thermal conductance k_e_, and the Seebeck coefficient S are shown in (**b**,**c**,**d**), respectively, at T = 5 K.

The effect of the QD length c on the electron transmission coefficient T_e_(E) and the electron transport coefficients G, k_e_, and S is shown in Figure 5 for variations in c in the range 10 nm–120 nm and constant QD width b = 20 nm.

**Figure 6:**
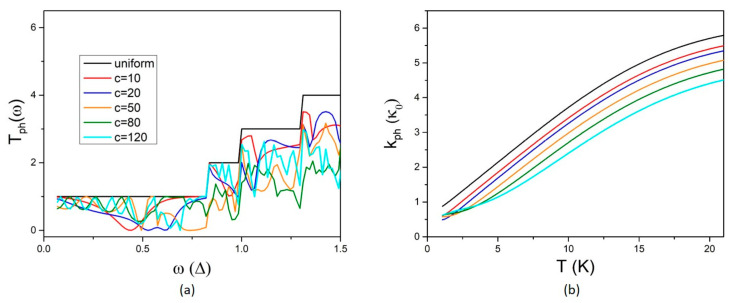
The effect of the QD length c on phonon transport: (**a**) the phonon transmission coefficient, T_ph_, and (**b**) the phonon thermal conductivity, k_ph_, versus temperature T for different values of the QD width b as denoted in the legend.

As expected, the characteristic non-monotonic fluctuations with peaks and dips are also present in this case as a signature of interference at the NW width discontinuities. Notably, a different behaviour can be observed now. Variation in the QD length c results in modifying the fluctuation spectrum rather than increasing the density of peaks and dips. The fluctuations in the electron transport properties clearly become denser with increasing QD length c up to the range c = 20–50 nm. For bigger values of c, the shape of the fluctuations changes while their density shows a weak modification. This can be more clearly shown in the Seebeck coefficient, which is more sensitive to the rate of variation as it is determined by the derivative of the energy fluctuations of T_e_. The Seebeck coefficient shows an increasing number of peaks with increasing c up to the range 20–50 nm and then the number and the height of the peaks remain nearly unaltered in the range c = 80–120 nm. The effect of the variation in the QD length c on phonon transport is shown in Figure 6. The phonon transmission coefficient shows denser fluctuations with increasing QD length c. Phonon thermal conductance decreases with increasing c. A weaker dependence can be observed at low temperatures, T < 5 K, which is attributed to the first NW sub-band being the only one that contributes to phonon transport at low temperatures.

**Figure 7:**
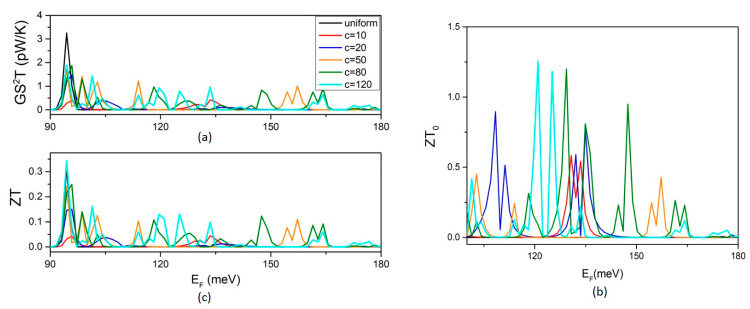
The effect of the QD length c on the thermoelectric efficiency: (**a**) the thermoelectric power factor TPF, (**b**) the optimal figure of merit ZT_0_, and (**c**) the figure of merit ZT at T = 5 K.

The effect of the variation in the QD length c on the TE efficiency is shown in Figure 7. The TPF shows similar dependences on the length c to G and S. The TPF peaks increase first with increasing length c in height and density. In the range 50–120 nm, the TPF peaks show nearly constant density and heights. The same conclusion can be drawn for the optimal figure of merit ZT_o_. The figure of merit ZT shows similar energy fluctuations to the TPF. The ZT peak’s height and density change slowly with c in the range 80–120 nm. A small increase in ZT can be noticed in this range due to the decrease in phonon thermal conductance with increasing width c (Figure 6b). It should be mentioned that further enhancement of ZT would be for width modulation via multiple QDs that would further decrease the phonon thermal conductance [11,12].

### 3.3. On the Range of Variation in b and c

The range of variation in the characteristic dimensions, b and c, was determined to provide (a) accurate results and (b) clear physics evidence. The accuracy of the results depends on the number of electron/phonon modes considered in the calculation of the corresponding scattering matrix elements, out of which the transmission coefficient is then calculated. When the size of a characteristic confinement dimension increases, the energy spectrum becomes denser and the number of contributing modes that are scattered at width discontinuities increases rapidly. Thus, quantum confinement dimensions must be kept within a range where calculations are feasible at an affordable computational cost. This sets limitations in the upper values of the characteristic lengths that can be modelled. In the present work, we extended the characteristic dimensions up to values where we could obtain clear physics evidence. The accuracy of the calculations was always checked and verified.

The range of variation in the length c of the QD is not restricted by the length of the NW. The lengths of the parts of the NWs on the left and on the right of the QD are infinite. Electron/phonon waves propagating in these parts of the MNW fall at width discontinuities where they are scattered and interfere with other scattered waves. Multiple scattering and quantum interference take place and determine the energy dependence of the electron/phonon transmission coefficient. As the length c increases, more wave modes are scattered, and the computation becomes harder. We limited our investigation up to c = 120 nm. Further increase in length c would add computational load without additional physics evidence.

Our study provides clear evidence that a decrease in the phonon thermal conductance with increasing QD length c would not necessarily increase ZT. This is due to the non-monotonic dependence of the thermoelectric power factor on the length c that we reveal. Figure 7a,b show that increasing c above 80 nm does not increase the TPF and the ZT_0_, respectively. A comparison of Figure 7a,c shows that the peaks of ZT are interpreted by the peaks of the TPF and thus it can be concluded that the ZT enhancement is dominated by the effect of width modulation on electrons. Figure 7c shows no significant enhancement in the height and density of the peaks of ZT with increasing c above 50 nm in the range 80–120 nm.

We studied the impact of width b and length c independently and successfully addressed the question of the effect of the width-mismatch on thermoelectric efficiency, revealing clear new evidence. Proceeding to explore combinations of values of b and c would not provide additional physical evidence or modify our conclusions. Such an investigation would serve the question of optimizing width-mismatch for maximum thermoelectric efficiency. This question is not at all trivial and is outside the scope of the present work. Our preliminary study shows that such optimization could not be unambiguously answered by identifying the set of b and c for the highest ZT. The interplay between b and c would not provide sufficient information for identifying a design strategy that would require optimization of the modulation profile [11,12,13]. Optimization of the modulation shape for maximum thermoelectric efficiency requires extended investigation with additional techniques such as machine learning, which is the subject of our ongoing investigation.

### 3.4. On Experimental Realization

We investigated MNWs of dimensions of the order of 10 nm that are feasible to fabricate and measure. Our study is in the ballistic transport regime where coherent carrier transport takes place. This regime is expected to dominate at low temperatures, typically below 10 K, because quantum confinement effects are screened via thermal broadening and scattering with increasing temperature. Coherent carrier (electron and phonon) transport in width-modulated nanowaveguides has been addressed in several theoretical studies (see Ref [1] for a review). However, we are not aware of any experimental study in this transport regime or on the thermoelectric efficiency of MNWs. Currently, there is increased research interest in coherent transport effects [31,32,33]. Coherent phonon transport has been confirmed in superlattices, nanomeshes, and holey nanobeams at low temperatures [33]. These findings call for further investigation and make research on coherent electron and phonon transmission across MNWs timely. Our results could serve to design experiments to investigate the effect of width-mismatch on the thermoelectric properties and efficiency of width-modulated nanowaveguides.

## 4. Conclusions

We addressed the problem of width-mismatch optimization for maximum thermoelectric efficiency. We explored the effect of changing the width-mismatch characteristic dimensions of a rectangular QD modulation unit and identified distinct behaviours for electrons and phonons. While phonon thermal conductance decreases with increasing width-mismatch, the electron thermoelectric power factor shows a non-monotonic dependence. Our results support that the optimal width-mismatch that maximizes thermoelectric efficiency is mainly determined by electron transport and can be identified by maximizing the thermoelectric power. This finding is important new information for the scientific community that currently focuses on increasing the thermoelectric efficiency by optimizing width modulation for minimum phonon conduction. Our work points to a new strategy of optimizing geometry-modulated metamaterials for maximum thermoelectric efficiency.

## Data Availability

Data are available by the authors upon reasonable request.
